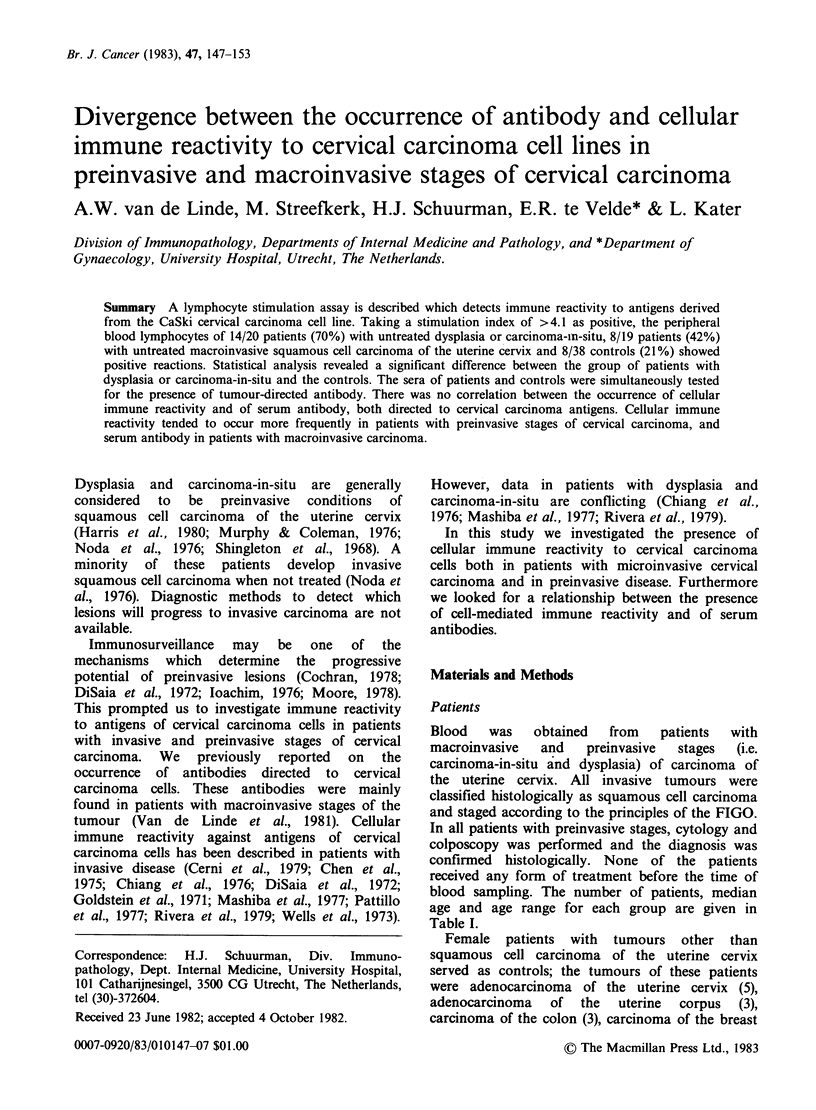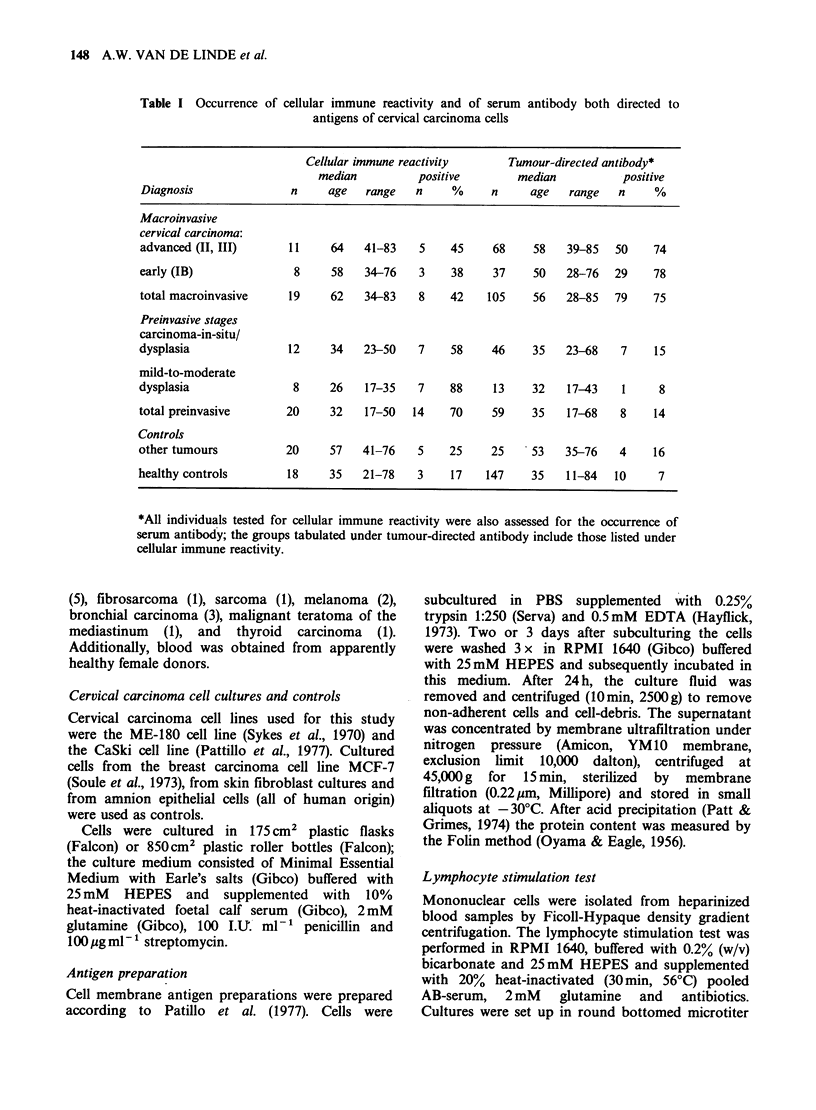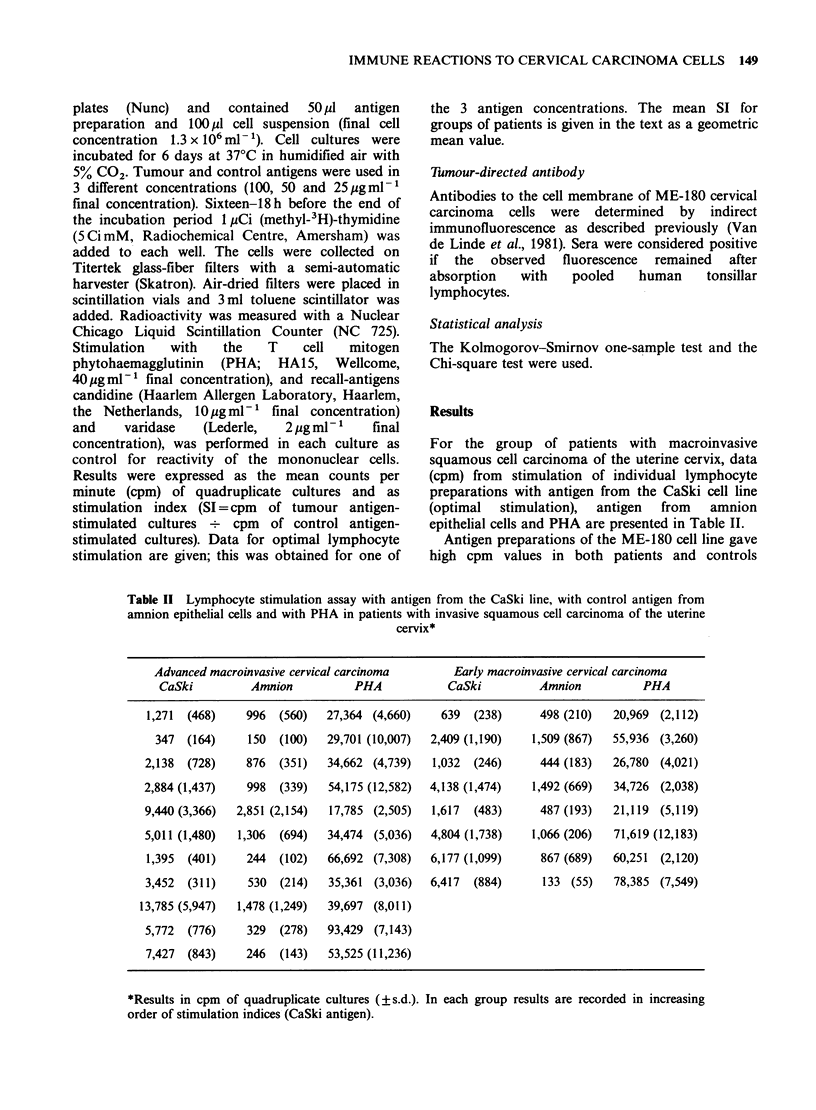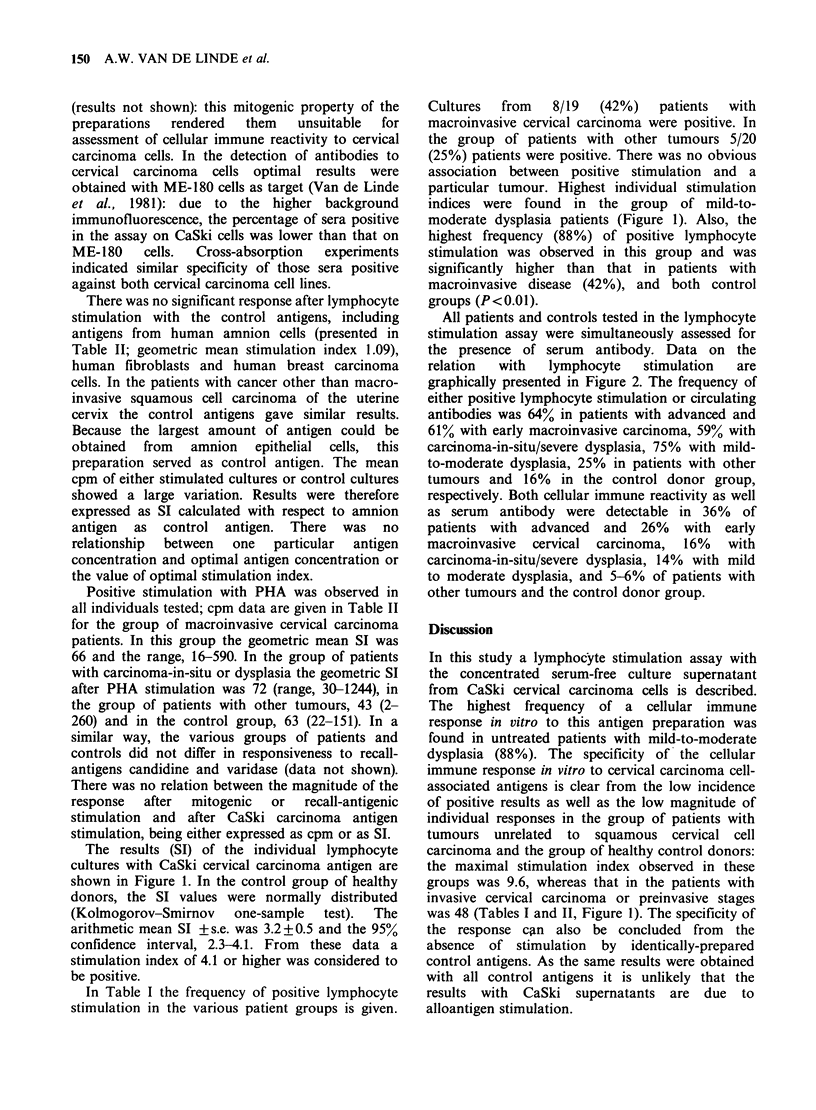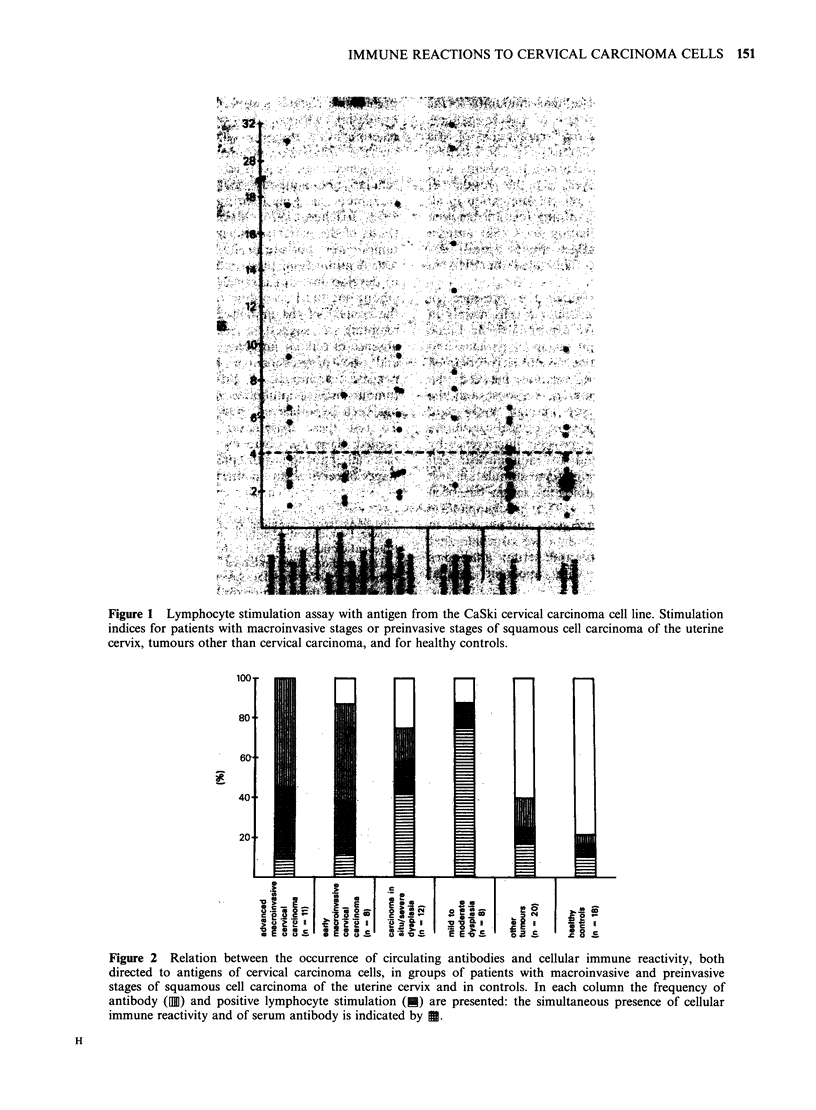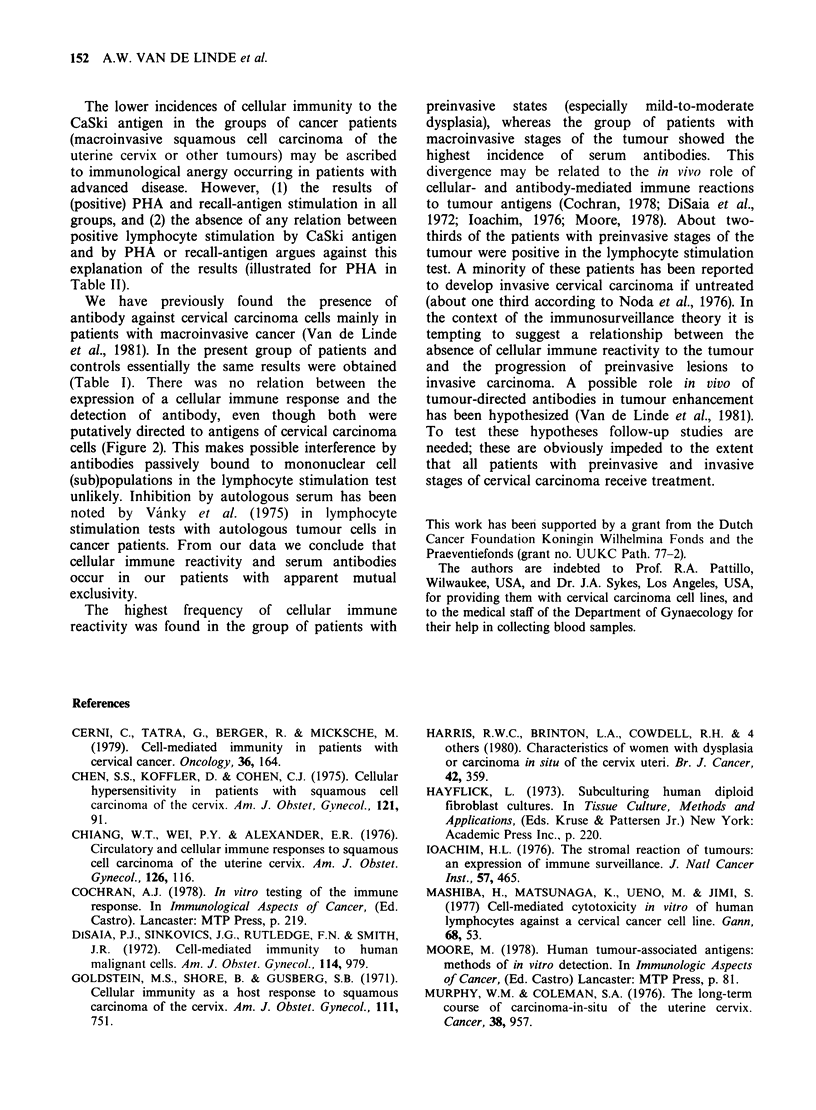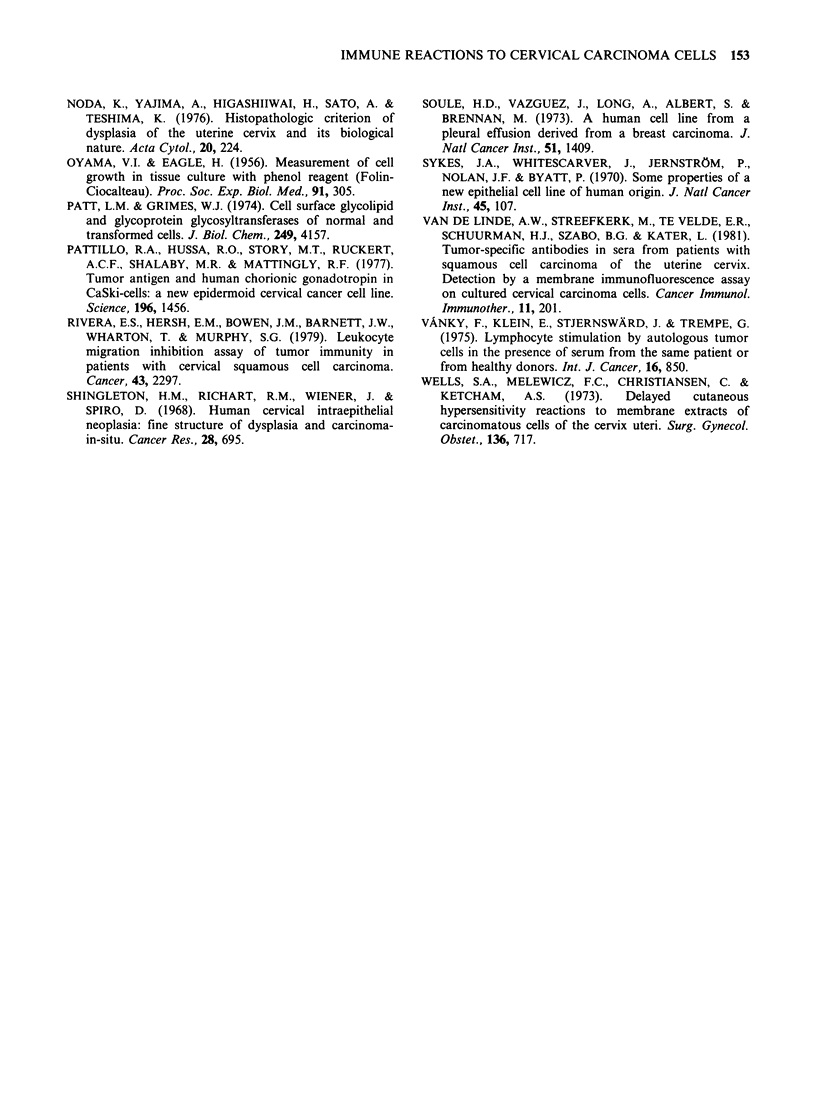# Divergence between the occurrence of antibody and cellular immune reactivity to cervical carcinoma cell lines in preinvasive and macroinvasive stages of cervical carcinoma.

**DOI:** 10.1038/bjc.1983.17

**Published:** 1983-01

**Authors:** A. W. van de Linde, M. Streefkerk, H. J. Schuurman, E. R. te Velde, L. Kater

## Abstract

A lymphocyte stimulation assay is described which detects immune reactivity to antigens derived from the CaSki cervical carcinoma cell line. Taking a stimulation index of greater than 4.1 as positive, the peripheral blood lymphocytes of 14/20 patients (70%) with untreated dysplasia or carcinoma-in-situ, 8/19 patients (42%) with untreated macroinvasive squamous cell carcinoma of the uterine cervix and 8/38 controls (21%) showed positive reactions. Statistical analysis revealed a significant difference between the group of patients with dysplasia or carcinoma-in-situ and the controls. The sera of patients and controls were simultaneously tested for the presence of tumour-directed antibody. There was no correlation between the occurrence of cellular immune reactivity and of serum antibody, both directed to cervical carcinoma antigens. Cellular immune reactivity tended to occur more frequently in patients with preinvasive stages of cervical carcinoma, and serum antibody in patients with macroinvasive carcinoma.


Br. J. Cancer (1983), 47, 147-153

### Divergence between the occurrence of antibody and cellular immune reactivity to cervical carcinoma cell lines in preinvasive and macroinvasive stages of cervical carcinoma

A.W. van de Linde, M. Streefkerk, H.J. Schuurman, E.R. te Velde* & L. Kater

*Division of Immunopathology, Departments of Internal Medicine and Pathology, and *Department of Gynaecology, University Hospital, Utrecht, The Netherlands.*

**Summary** A lymphocyte stimulation assay is described which detects immune reactivity to antigens derived from the CaSki cervical carcinoma cell line. Taking a stimulation index of >4.1 as positive, the peripheral blood lymphocytes of 14/20 patients (70%) with untreated dysplasia or carcinoma-in-situ, 8/19 patients (42%) with untreated macroinvasive squamous cell carcinoma of the uterine cervix and 8/38 controls (21%) showed positive reactions. Statistical analysis revealed a significant difference between the group of patients with dysplasia or carcinoma-in-situ and the controls. The sera of patients and controls were simultaneously tested for the presence of tumour-directed antibody. There was no correlation between the occurrence of cellular immune reactivity and of serum antibody, both directed to cervical carcinoma antigens. Cellular immune reactivity tended to occur more frequently in patients with preinvasive stages of cervical carcinoma, and serum antibody in patients with macroinvasive carcinoma.

Dysplasia and carcinoma-in-situ are generally considered to be preinvasive conditions of squamous cell carcinoma of the uterine cervix (Harris *et al.*, 1980; Murphy & Coleman, 1976; Noda *et al.*, 1976; Shingleton *et al.*, 1968). A minority of these patients develop invasive squamous cell carcinoma when not treated (Noda *et al.*, 1976). Diagnostic methods to detect which lesions will progress to invasive carcinoma are not available.

Immunosurveillance may be one of the mechanisms which determine the progressive potential of preinvasive lesions (Cochran, 1978; DiSaia *et al.*, 1972; Ioachim, 1976; Moore, 1978). This prompted us to investigate immune reactivity to antigens of cervical carcinoma cells in patients with invasive and preinvasive stages of cervical carcinoma. We previously reported on the occurrence of antibodies directed to cervical carcinoma cells. These antibodies were mainly found in patients with macroinvasive stages of the tumour (Van de Linde *et al.*, 1981). Cellular immune reactivity against antigens of cervical carcinoma cells has been described in patients with invasive disease (Cerni *et al.*, 1979; Chen *et al.*, 1975; Chiang *et al.*, 1976; DiSaia *et al.*, 1972; Goldstein *et al.*, 1971; Mashiba *et al.*, 1977; Pattillo *et al.*, 1977; Rivera *et al.*, 1979; Wells *et al.*, 1973). However, data in patients with dysplasia and carcinoma-in-situ are conflicting (Chiang *et al.*, 1976; Mashiba *et al.*, 1977; Rivera *et al.*, 1979).

In this study we investigated the presence of cellular immune reactivity to cervical carcinoma cells both in patients with microinvasive cervical carcinoma and in preinvasive disease. Furthermore we looked for a relationship between the presence of cell-mediated immune reactivity and of serum antibodies.

### Materials and Methods

### *Patients*

Blood was obtained from patients with macroinvasive and preinvasive stages (i.e. carcinoma-in-situ and dysplasia) of carcinoma of the uterine cervix. All invasive tumours were classified histologically as squamous cell carcinoma and staged according to the principles of the FIGO. In all patients with preinvasive stages, cytology and colposcopy was performed and the diagnosis was confirmed histologically. None of the patients received any form of treatment before the time of blood sampling. The number of patients, median age and age range for each group are given in Table I.

Female patients with tumours other than squamous cell carcinoma of the uterine cervix served as controls; the tumours of these patients were adenocarcinoma of the uterine cervix (5), adenocarcinoma of the uterine corpus (3), carcinoma of the colon (3), carcinoma of the breast

Correspondence: H.J. Schuurman, Div. Immunopathology, Dept. Internal Medicine, University Hospital, 101 Catharijnesingel, 3500 CG Utrecht, The Netherlands, tel (30)-372604.

Received 23 June 1982; accepted 4 October 1982.

0007-0920/83/010147-07 $01.00

© The Macmillan Press Ltd., 1983

**Table I** Occurrence of cellular immune reactivity and of serum antibody both directed to antigens of cervical carcinoma cells

| | *Cellular immune reactivity* | | | | | *Tumour-directed antibody** | | | | |
|---|---|---|---|---|---|---|---|---|---|---|
| | | *median* | | *positive* | | | *median* | | *positive* | |
| *Diagnosis* | *n* | *age* | *range* | *n* | *%* | *n* | *age* | *range* | *n* | *%* |
| *Macroinvasive cervical carcinoma*: | | | | | | | | | | |
| advanced (II, III) | 11 | 64 | 41–83 | 5 | 45 | 68 | 58 | 39–85 | 50 | 74 |
| early (IB) | 8 | 58 | 34–76 | 3 | 38 | 37 | 50 | 28–76 | 29 | 78 |
| total macroinvasive | 19 | 62 | 34–83 | 8 | 42 | 105 | 56 | 28–85 | 79 | 75 |
| *Preinvasive stages* | | | | | | | | | | |
| carcinoma-in-situ/ dysplasia | 12 | 34 | 23–50 | 7 | 58 | 46 | 35 | 23–68 | 7 | 15 |
| mild-to-moderate dysplasia | 8 | 26 | 17–35 | 7 | 88 | 13 | 32 | 17–43 | 1 | 8 |
| total preinvasive | 20 | 32 | 17–50 | 14 | 70 | 59 | 35 | 17–68 | 8 | 14 |
| *Controls* | | | | | | | | | | |
| other tumours | 20 | 57 | 41–76 | 5 | 25 | 25 | 53 | 35–76 | 4 | 16 |
| healthy controls | 18 | 35 | 21–78 | 3 | 17 | 147 | 35 | 11–84 | 10 | 7 |

*All individuals tested for cellular immune reactivity were also assessed for the occurrence of serum antibody; the groups tabulated under tumour-directed antibody include those listed under cellular immune reactivity.

(5), fibrosarcoma (1), sarcoma (1), melanoma (2), bronchial carcinoma (3), malignant teratoma of the mediastinum (1), and thyroid carcinoma (1). Additionally, blood was obtained from apparently healthy female donors.

### *Cervical carcinoma cell cultures and controls*

Cervical carcinoma cell lines used for this study were the ME-180 cell line (Sykes *et al.*, 1970) and the CaSki cell line (Pattillo *et al.*, 1977). Cultured cells from the breast carcinoma cell line MCF-7 (Soule *et al.*, 1973), from skin fibroblast cultures and from amnion epithelial cells (all of human origin) were used as controls.

Cells were cultured in 175 $cm^2$ plastic flasks (Falcon) or 850 $cm^2$ plastic roller bottles (Falcon); the culture medium consisted of Minimal Essential Medium with Earle's salts (Gibco) buffered with 25 mM HEPES and supplemented with 10% heat-inactivated foetal calf serum (Gibco), 2 mM glutamine (Gibco), 100 I.U. $ml^{-1}$ penicillin and 100 $\mu g\, ml^{-1}$ streptomycin.

### *Antigen preparation*

Cell membrane antigen preparations were prepared according to Patillo *et al.* (1977). Cells were subcultured in PBS supplemented with 0.25% trypsin 1:250 (Serva) and 0.5 mM EDTA (Hayflick, 1973). Two or 3 days after subculturing the cells were washed 3 × in RPMI 1640 (Gibco) buffered with 25 mM HEPES and subsequently incubated in this medium. After 24 h, the culture fluid was removed and centrifuged (10 min, 2500 g) to remove non-adherent cells and cell-debris. The supernatant was concentrated by membrane ultrafiltration under nitrogen pressure (Amicon, YM10 membrane, exclusion limit 10,000 dalton), centrifuged at 45,000 g for 15 min, sterilized by membrane filtration (0.22 $\mu$m, Millipore) and stored in small aliquots at −30°C. After acid precipitation (Patt & Grimes, 1974) the protein content was measured by the Folin method (Oyama & Eagle, 1956).

### *Lymphocyte stimulation test*

Mononuclear cells were isolated from heparinized blood samples by Ficoll-Hypaque density gradient centrifugation. The lymphocyte stimulation test was performed in RPMI 1640, buffered with 0.2% (w/v) bicarbonate and 25 mM HEPES and supplemented with 20% heat-inactivated (30 min, 56°C) pooled AB-serum, 2 mM glutamine and antibiotics. Cultures were set up in round bottomed microtiter plates (Nunc) and contained 50 $\mu$l antigen preparation and 100 $\mu$l cell suspension (final cell concentration $1.3 \times 10^6\,ml^{-1}$). Cell cultures were incubated for 6 days at 37°C in humidified air with 5% $CO_2$. Tumour and control antigens were used in 3 different concentrations (100, 50 and 25 $\mu g\,ml^{-1}$ final concentration). Sixteen–18 h before the end of the incubation period 1 $\mu$Ci (methyl-$^3$H)-thymidine (5 Ci mM, Radiochemical Centre, Amersham) was added to each well. The cells were collected on Titertek glass-fiber filters with a semi-automatic harvester (Skatron). Air-dried filters were placed in scintillation vials and 3 ml toluene scintillator was added. Radioactivity was measured with a Nuclear Chicago Liquid Scintillation Counter (NC 725). Stimulation with the T cell mitogen phytohaemagglutinin (PHA; HA15, Wellcome, 40 $\mu g\,ml^{-1}$ final concentration), and recall-antigens candidine (Haarlem Allergen Laboratory, Haarlem, the Netherlands, 10 $\mu g\,ml^{-1}$ final concentration) and varidase (Lederle, 2 $\mu g\,ml^{-1}$ final concentration), was performed in each culture as control for reactivity of the mononuclear cells. Results were expressed as the mean counts per minute (cpm) of quadruplicate cultures and as stimulation index (SI = cpm of tumour antigen-stimulated cultures ÷ cpm of control antigen-stimulated cultures). Data for optimal lymphocyte stimulation are given; this was obtained for one of the 3 antigen concentrations. The mean SI for groups of patients is given in the text as a geometric mean value.

### *Tumour-directed antibody*

Antibodies to the cell membrane of ME-180 cervical carcinoma cells were determined by indirect immunofluorescence as described previously (Van de Linde *et al.*, 1981). Sera were considered positive if the observed fluorescence remained after absorption with pooled human tonsillar lymphocytes.

### *Statistical analysis*

The Kolmogorov–Smirnov one-sample test and the Chi-square test were used.

### Results

For the group of patients with macroinvasive squamous cell carcinoma of the uterine cervix, data (cpm) from stimulation of individual lymphocyte preparations with antigen from the CaSki cell line (optimal stimulation), antigen from amnion epithelial cells and PHA are presented in Table II.

Antigen preparations of the ME-180 cell line gave high cpm values in both patients and controls

**Table II** Lymphocyte stimulation assay with antigen from the CaSki line, with control antigen from amnion epithelial cells and with PHA in patients with invasive squamous cell carcinoma of the uterine cervix*

| *Advanced macroinvasive cervical carcinoma* | | | *Early macroinvasive cervical carcinoma* | | |
|---|---|---|---|---|---|
| *CaSki* | *Amnion* | *PHA* | *CaSki* | *Amnion* | *PHA* |
| 1,271 (468) | 996 (560) | 27,364 (4,660) | 639 (238) | 498 (210) | 20,969 (2,112) |
| 347 (164) | 150 (100) | 29,701 (10,007) | 2,409 (1,190) | 1,509 (867) | 55,936 (3,260) |
| 2,138 (728) | 876 (351) | 34,662 (4,739) | 1,032 (246) | 444 (183) | 26,780 (4,021) |
| 2,884 (1,437) | 998 (339) | 54,175 (12,582) | 4,138 (1,474) | 1,492 (669) | 34,726 (2,038) |
| 9,440 (3,366) | 2,851 (2,154) | 17,785 (2,505) | 1,617 (483) | 487 (193) | 21,119 (5,119) |
| 5,011 (1,480) | 1,306 (694) | 34,474 (5,036) | 4,804 (1,738) | 1,066 (206) | 71,619 (12,183) |
| 1,395 (401) | 244 (102) | 66,692 (7,308) | 6,177 (1,099) | 867 (689) | 60,251 (2,120) |
| 3,452 (311) | 530 (214) | 35,361 (3,036) | 6,417 (884) | 133 (55) | 78,385 (7,549) |
| 13,785 (5,947) | 1,478 (1,249) | 39,697 (8,011) | | | |
| 5,772 (776) | 329 (278) | 93,429 (7,143) | | | |
| 7,427 (843) | 246 (143) | 53,525 (11,236) | | | |

*Results in cpm of quadruplicate cultures (±s.d.). In each group results are recorded in increasing order of stimulation indices (CaSki antigen).

(results not shown): this mitogenic property of the preparations rendered them unsuitable for assessment of cellular immune reactivity to cervical carcinoma cells. In the detection of antibodies to cervical carcinoma cells optimal results were obtained with ME-180 cells as target (Van de Linde *et al.*, 1981): due to the higher background immunofluorescence, the percentage of sera positive in the assay on CaSki cells was lower than that on ME-180 cells. Cross-absorption experiments indicated similar specificity of those sera positive against both cervical carcinoma cell lines.

There was no significant response after lymphocyte stimulation with the control antigens, including antigens from human amnion cells (presented in Table II; geometric mean stimulation index 1.09), human fibroblasts and human breast carcinoma cells. In the patients with cancer other than macro-invasive squamous cell carcinoma of the uterine cervix the control antigens gave similar results. Because the largest amount of antigen could be obtained from amnion epithelial cells, this preparation served as control antigen. The mean cpm of either stimulated cultures or control cultures showed a large variation. Results were therefore expressed as SI calculated with respect to amnion antigen as control antigen. There was no relationship between one particular antigen concentration and optimal antigen concentration or the value of optimal stimulation index.

Positive stimulation with PHA was observed in all individuals tested; cpm data are given in Table II for the group of macroinvasive cervical carcinoma patients. In this group the geometric mean SI was 66 and the range, 16–590. In the group of patients with carcinoma-in-situ or dysplasia the geometric SI after PHA stimulation was 72 (range, 30–1244), in the group of patients with other tumours, 43 (2–260) and in the control group, 63 (22–151). In a similar way, the various groups of patients and controls did not differ in responsiveness to recall-antigens candidine and varidase (data not shown). There was no relation between the magnitude of the response after mitogenic or recall-antigenic stimulation and after CaSki carcinoma antigen stimulation, being either expressed as cpm or as SI.

The results (SI) of the individual lymphocyte cultures with CaSki cervical carcinoma antigen are shown in Figure 1. In the control group of healthy donors, the SI values were normally distributed (Kolmogorov–Smirnov one-sample test). The arithmetic mean SI $\pm$s.e. was $3.2 \pm 0.5$ and the 95% confidence interval, 2.3–4.1. From these data a stimulation index of 4.1 or higher was considered to be positive.

In Table I the frequency of positive lymphocyte stimulation in the various patient groups is given. Cultures from 8/19 (42%) patients with macroinvasive cervical carcinoma were positive. In the group of patients with other tumours 5/20 (25%) patients were positive. There was no obvious association between positive stimulation and a particular tumour. Highest individual stimulation indices were found in the group of mild-to-moderate dysplasia patients (Figure 1). Also, the highest frequency (88%) of positive lymphocyte stimulation was observed in this group and was significantly higher than that in patients with macroinvasive disease (42%), and both control groups ($P < 0.01$).

All patients and controls tested in the lymphocyte stimulation assay were simultaneously assessed for the presence of serum antibody. Data on the relation with lymphocyte stimulation are graphically presented in Figure 2. The frequency of either positive lymphocyte stimulation or circulating antibodies was 64% in patients with advanced and 61% with early macroinvasive carcinoma, 59% with carcinoma-in-situ/severe dysplasia, 75% with mild-to-moderate dysplasia, 25% in patients with other tumours and 16% in the control donor group, respectively. Both cellular immune reactivity as well as serum antibody were detectable in 36% of patients with advanced and 26% with early macroinvasive cervical carcinoma, 16% with carcinoma-in-situ/severe dysplasia, 14% with mild to moderate dysplasia, and 5–6% of patients with other tumours and the control donor group.

### Discussion

In this study a lymphocyte stimulation assay with the concentrated serum-free culture supernatant from CaSki cervical carcinoma cells is described. The highest frequency of a cellular immune response *in vitro* to this antigen preparation was found in untreated patients with mild-to-moderate dysplasia (88%). The specificity of the cellular immune response *in vitro* to cervical carcinoma cell-associated antigens is clear from the low incidence of positive results as well as the low magnitude of individual responses in the group of patients with tumours unrelated to squamous cervical cell carcinoma and the group of healthy control donors: the maximal stimulation index observed in these groups was 9.6, whereas that in the patients with invasive cervical carcinoma or preinvasive stages was 48 (Tables I and II, Figure 1). The specificity of the response can also be concluded from the absence of stimulation by identically-prepared control antigens. As the same results were obtained with all control antigens it is unlikely that the results with CaSki supernatants are due to alloantigen stimulation.

**Figure 1** Lymphocyte stimulation assay with antigen from the CaSki cervical carcinoma cell line. Stimulation indices for patients with macroinvasive stages or preinvasive stages of squamous cell carcinoma of the uterine cervix, tumours other than cervical carcinoma, and for healthy controls.

**Figure 2** Relation between the occurrence of circulating antibodies and cellular immune reactivity, both directed to antigens of cervical carcinoma cells, in groups of patients with macroinvasive and preinvasive stages of squamous cell carcinoma of the uterine cervix and in controls. In each column the frequency of antibody (▥) and positive lymphocyte stimulation (▤) are presented: the simultaneous presence of cellular immune reactivity and of serum antibody is indicated by ▦.

The lower incidences of cellular immunity to the CaSki antigen in the groups of cancer patients (macroinvasive squamous cell carcinoma of the uterine cervix or other tumours) may be ascribed to immunological anergy occurring in patients with advanced disease. However, (1) the results of (positive) PHA and recall-antigen stimulation in all groups, and (2) the absence of any relation between positive lymphocyte stimulation by CaSki antigen and by PHA or recall-antigen argues against this explanation of the results (illustrated for PHA in Table II).

We have previously found the presence of antibody against cervical carcinoma cells mainly in patients with macroinvasive cancer (Van de Linde *et al.*, 1981). In the present group of patients and controls essentially the same results were obtained (Table I). There was no relation between the expression of a cellular immune response and the detection of antibody, even though both were putatively directed to antigens of cervical carcinoma cells (Figure 2). This makes possible interference by antibodies passively bound to mononuclear cell (sub)populations in the lymphocyte stimulation test unlikely. Inhibition by autologous serum has been noted by Vánky *et al.* (1975) in lymphocyte stimulation tests with autologous tumour cells in cancer patients. From our data we conclude that cellular immune reactivity and serum antibodies occur in our patients with apparent mutual exclusivity.

The highest frequency of cellular immune reactivity was found in the group of patients with preinvasive states (especially mild-to-moderate dysplasia), whereas the group of patients with macroinvasive stages of the tumour showed the highest incidence of serum antibodies. This divergence may be related to the *in vivo* role of cellular- and antibody-mediated immune reactions to tumour antigens (Cochran, 1978; DiSaia *et al.*, 1972; Ioachim, 1976; Moore, 1978). About two-thirds of the patients with preinvasive stages of the tumour were positive in the lymphocyte stimulation test. A minority of these patients has been reported to develop invasive cervical carcinoma if untreated (about one third according to Noda *et al.*, 1976). In the context of the immunosurveillance theory it is tempting to suggest a relationship between the absence of cellular immune reactivity to the tumour and the progression of preinvasive lesions to invasive carcinoma. A possible role *in vivo* of tumour-directed antibodies in tumour enhancement has been hypothesized (Van de Linde *et al.*, 1981). To test these hypotheses follow-up studies are needed; these are obviously impeded to the extent that all patients with preinvasive and invasive stages of cervical carcinoma receive treatment.

This work has been supported by a grant from the Dutch Cancer Foundation Koningin Wilhelmina Fonds and the Praeventiefonds (grant no. UUKC Path. 77–2).

The authors are indebted to Prof. R.A. Pattillo, Wilwaukee, USA, and Dr. J.A. Sykes, Los Angeles, USA, for providing them with cervical carcinoma cell lines, and to the medical staff of the Department of Gynaecology for their help in collecting blood samples.

### References

CERNI, C., TATRA, G., BERGER, R. & MICKSCHE, M. (1979). Cell-mediated immunity in patients with cervical cancer. *Oncology*, **36**, 164.

CHEN, S.S., KOFFLER, D. & COHEN, C.J. (1975). Cellular hypersensitivity in patients with squamous cell carcinoma of the cervix. *Am. J. Obstet, Gynecol.*, **121**, 91.

CHIANG, W.T., WEI, P.Y. & ALEXANDER, E.R. (1976). Circulatory and cellular immune responses to squamous cell carcinoma of the uterine cervix. *Am. J. Obstet. Gynecol.*, **126**, 116.

COCHRAN, A.J. (1978). *In vitro* testing of the immune response. In *Immunological Aspects of Cancer*, (Ed. Castro). Lancaster: MTP Press, p. 219.

DISAIA, P.J., SINKOVICS, J.G., RUTLEDGE, F.N. & SMITH, J.R. (1972). Cell-mediated immunity to human malignant cells. *Am. J. Obstet. Gynecol.*, **114**, 979.

GOLDSTEIN, M.S., SHORE, B. & GUSBERG, S.B. (1971). Cellular immunity as a host response to squamous carcinoma of the cervix. *Am. J. Obstet. Gynecol.*, **111**, 751.

HARRIS, R.W.C., BRINTON, L.A., COWDELL, R.H. & 4 others (1980). Characteristics of women with dysplasia or carcinoma *in situ* of the cervix uteri. *Br. J. Cancer*, **42**, 359.

HAYFLICK, L. (1973). Subculturing human diploid fibroblast cultures. In *Tissue Culture, Methods and Applications*, (Eds. Kruse & Pattersen Jr.) New York: Academic Press Inc., p. 220.

IOACHIM, H.L. (1976). The stromal reaction of tumours: an expression of immune surveillance. *J. Natl Cancer Inst.*, **57**, 465.

MASHIBA, H., MATSUNAGA, K., UENO, M. & JIMI, S. (1977) Cell-mediated cytotoxicity *in vitro* of human lymphocytes against a cervical cancer cell line. *Gann*, **68**, 53.

MOORE, M. (1978). Human tumour-associated antigens: methods of *in vitro* detection. In *Immunologic Aspects of Cancer*, (Ed. Castro) Lancaster: MTP Press, p. 81.

MURPHY, W.M. & COLEMAN, S.A. (1976). The long-term course of carcinoma-in-situ of the uterine cervix. *Cancer*, **38**, 957.

NODA, K., YAJIMA, A., HIGASHIIWAI, H., SATO, A. & TESHIMA, K. (1976). Histopathologic criterion of dysplasia of the uterine cervix and its biological nature. *Acta Cytol.*, **20,** 224.

OYAMA, V.I. & EAGLE, H. (1956). Measurement of cell growth in tissue culture with phenol reagent (Folin-Ciocalteau). *Proc. Soc. Exp. Biol. Med.*, **91,** 305.

PATT, L.M. & GRIMES, W.J. (1974). Cell surface glycolipid and glycoprotein glycosyltransferases of normal and transformed cells. *J. Biol. Chem.*, **249,** 4157.

PATTILLO, R.A., HUSSA, R.O., STORY, M.T., RUCKERT, A.C.F., SHALABY, M.R. & MATTINGLY, R.F. (1977). Tumor antigen and human chorionic gonadotropin in CaSki-cells: a new epidermoid cervical cancer cell line. *Science*, **196,** 1456.

RIVERA, E.S., HERSH, E.M., BOWEN, J.M., BARNETT, J.W., WHARTON, T. & MURPHY, S.G. (1979). Leukocyte migration inhibition assay of tumor immunity in patients with cervical squamous cell carcinoma. *Cancer*, **43,** 2297.

SHINGLETON, H.M., RICHART, R.M., WIENER, J. & SPIRO, D. (1968). Human cervical intraepithelial neoplasia: fine structure of dysplasia and carcinoma-in-situ. *Cancer Res.*, **28,** 695.

SOULE, H.D., VAZGUEZ, J., LONG, A., ALBERT, S. & BRENNAN, M. (1973). A human cell line from a pleural effusion derived from a breast carcinoma. *J. Natl Cancer Inst.*, **51,** 1409.

SYKES, J.A., WHITESCARVER, J., JERNSTRÖM, P., NOLAN, J.F. & BYATT, P. (1970). Some properties of a new epithelial cell line of human origin. *J. Natl Cancer Inst.*, **45,** 107.

VAN DE LINDE, A.W., STREEFKERK, M., TE VELDE, E.R., SCHUURMAN, H.J., SZABO, B.G. & KATER, L. (1981). Tumor-specific antibodies in sera from patients with squamous cell carcinoma of the uterine cervix. Detection by a membrane immunofluorescence assay on cultured cervical carcinoma cells. *Cancer Immunol. Immunother.*, **11,** 201.

VÁNKY, F., KLEIN, E., STJERNSWÄRD, J. & TREMPE, G. (1975). Lymphocyte stimulation by autologous tumor cells in the presence of serum from the same patient or from healthy donors. *Int. J. Cancer*, **16,** 850.

WELLS, S.A., MELEWICZ, F.C., CHRISTIANSEN, C. & KETCHAM, A.S. (1973). Delayed cutaneous hypersensitivity reactions to membrane extracts of carcinomatous cells of the cervix uteri. *Surg. Gynecol. Obstet.*, **136,** 717.